# Comparison of frozen-thawed embryo transfer protocols in patients with previous cycle cancellation due to uterine peristalsis: a pilot study

**DOI:** 10.3906/sag-2012-149

**Published:** 2021-06-28

**Authors:** İlknur SELVİ, Mehmet ERDEM, Erhan DEMİRDAĞ, Funda CEVHER, Cengiz KARAKAYA, Ahmet ERDEM

**Affiliations:** 1 Department of Obstetrics & Gynecology, Koru Hospital, Ankara Turkey; 2 Department of Obstetrics & Gynecology, Faculty of Medicine, Gazi University, Ankara Turkey; 3 Department of Medical Biochemistry and IVF Unit, Faculty of Medicine, Gazi University, Ankara Turkey

**Keywords:** Uterine peristalsis, frozen-thawed cycle, embryo transfer, letrozole, atosiban

## Abstract

**Background/aim:**

To investigate the optimal protocol for frozen-thawed embryo transfer (FET) cycles in patients who previously had a cycle cancellation due to uterine peristalsis (UP).

**Materials and methods:**

Thirty-four patients with previous embryo transfer (ET) cancellation due to UP during artificial cycle (AC) were included retrospectively. In the proceeding cycle, endometrium was prepared with AC (n: 23) in AC-FET group or with stimulated cycle that contains letrozole (L) (n: 11) in L-FET group. Intravenous bolus dose of 6.75 mg atosiban (Tractocile; Ferring Pharmaceuticals, Switzerland) injection was performed to all patients of AC-FET group due to UP ≥ 4/min on the planned ET day of proceeding cycle. Atosiban was not used in L-FET group. Primary outcome was live birth rate (LBR) per ET. Secondary outcomes were clinical pregnancy rate (CPR) per ET, implantation rate (IR), cycle cancellation rate.

**Results:**

The baseline characteristics such as age, body mass index, antral follicle count, duration of infertility, and the number of prior in vitro fertilization attempts of each group were similar. The IR, CPR per ET, LBR per ET, CPR per cycle and LBR per cycle were significantly higher; cycle cancellation rates were significantly lower in L-FET group as compared to the AC-FET group.

**Conclusion:**

Endometrial preparation with letrozole significantly improves CPR and LBR in FET cycles of patients with previous cycle cancellations due to UP.

## 1. Introduction

Frozen-thawed embryo transfer (FET) has gained importance in assisted reproduction technology (ART) worldwide with the improvements in the vitrification techniques and increased pregnancy rates [1]. It gives the opportunity of storing surplus embryos and transferring them in more suitable conditions especially in cases of increased risk of ovarian hyperstimulation syndrome (OHSS) [2] and in the presence of endometrial disturbances, with lower cost and simpler than repeated fresh cycles [3].

Despite the improvements in ART, in vitro fertilization (IVF) success rate in terms of live birth per cycle is 26.9% [4]. Two important factors that affect implantation and consequently IVF outcome are embryo quality and endometrial receptivity [5–9]. Endometrial thickness and pattern should be appropriate and blood flow should be sufficient for a receptive endometrium [8,10–12]. Endometrial receptivity and implantation has been impaired in a stimulated cycle as a result of supraphysiological endocrine milieu [13–15].

Ovarian stimulation cycle differs from a natural cycle by means of endocrine milieu with low progesterone [16] and supraphysiological level of estradiol [17]. Elevated serum estradiol has deleterious effects on endometrium by changing protein expressions [18]. It also effects directly embryo and disrupts embryo adhesion [19]. Another undesirable effect of ovarian stimulation is thought to be increase in uterine activity due to exaggerated oxytocin action stimulated by elevated levels of estradiol [20]. It was shown that expressions of uterine oxytocin mRNA and uterine oxytocin receptors were ovarian hormone-dependent. Oxytocin produced by endometrium is highest in the midcycle in natural cycles [21,22] to stimulate myometrial activity of nonpregnant uterus and plays a role in sperm and egg transport and implantation [23]. It is also increased 300-fold during pregnancy as a stimulatory effect of estrogens [24]. 

Uterine peristalsis (UP) was detected in 30% of patients before embryo transfer (ET) [25] and implantation rates in cases of UP were 3-fold lower compared with silent uteri [26]. Several medical treatments have been used for UP treatment [27]. Due to the lack of large sample sized randomized controlled studies, to date there is not any confirmed diagnostic tool and therapeutic approach for UP [8].

As with the treatment of UP, there are also controversies regarding the best FET protocol [1,28]. The protocols used for FET are natural cycle, modified natural cycle, artificial cycle (AC-FET) with or without suppression, mild ovarian stimulation with gonadotropins and aromatase inhibitors as letrozole [1]. Letrozole, third generation aromatase inhibitor, is a short half-life agent for ovulation induction that leads generally to monofollicular development with maintenance of normal central feedback and not blocking the estrogen receptors [29]. It was shown that letrozole increased endometrial receptivity markers [30]. While letrozole inhibits the conversion of androgen to estradiol and causes much lower estradiol levels than the natural cycle, it does not cause negative effects on endometrial thickness [6]. Letrozole was indirectly used for endometrial preparation in FET cycles of patients with polycystic ovary syndrome (PCOS) [6,29,31].

The aim of the study was to investigate the effects of letrozole-stimulated endometrial preparation in the patients with a history of UP and to compare the pregnancy outcomes of L-FET and AC-FET cycles in these patients. The hypothesis was that letrozole usage for endometrial preparation can lead to lower estradiol levels and decreased production of endometrial oxytocin, which triggers UP. To the best of our knowledge, this is the first study that compares AC and letrozole-stimulated cycle in patients with previous cycle cancellation due to UP.

## 2. Materials and methods

Patients whose embryo transfers (ET) were cancelled in previous AC-FET cycle due to uterine contractions (UC) ≥ 4/min that were realized on routine transvaginal ultrasonography (TVUSG) controls and persisted on planned ET day were included in this retrospective pilot study. All included patients were treated for a FET cycle from January 2019 to May 2019 in NovaArt IVF Center. Considering the option of administering atosiban if UP recurs, AC was restarted in one group; while the other group was given letrozole for endometrial preparation. Exclusion criteria were the women aged >42 years, antral follicle count (AFC) < 5, uterine pathologies as adenomyosis, uterine anomaly, uterine fibroids, and hydrosalpinges. Embryo quality was assessed after warming according to the number and regularity of blastomeres and the degree of fragmentation. The institutional review board and ethics committee approved the study.

In L-FET group, endometrium was prepared with letrozole (Femara; Novartis, Switzerland) 5 mg daily for 5 days beginning on day 3 of menses. When preovulatory follicle reached 17 mm; serum E2, LH, and progesterone levels were checked every day. When endometrial thickness was ≥7 mm and serum E2 level was >150 pg/mL with the presence of corpus luteum or in the case of corpus luteum uncertainty in TVUSG if progesterone level above 1.5 ng/mL was detected after LH peak, vaginal progesterone (400 mg/day; Progestan; Kocak Farma, Turkey) was started. 

In AC-FET protocol, endometrium was prepared with estradiol valerate 2 mg three times daily (Estrofem; Novo Nordisk, Turkey) beginning on day 3 of menses. If endometrial thickness was ≥7 mm and serum E2 level ws >150 pg/mL, vaginal progesterone (400 mg/day; Progestan; Kocak Farma, Turkey) was started. In both protocol, ET was scheduled on the 4th day for cleavage embryo or 6th day for blastocyst. Luteal support was continued until 10 weeks of gestation if pregnancy occurs. 

Endometrial thickness and pattern were evaluated with TVUSG (Aloka SSD-1000, Japan) with a 5 MHz transvaginal probe during routine examinations. The number of UP per minute was calculated by directly counting the peristaltic waveforms seen in the endometrium in the sagittal plane on transvaginal ultrasound examination for 1 min. All TVUSG examinations were performed with the same single sonographer (I.S). The patients that had UC ≥ 4/min were examined with TVUSG on the planned ET day again. According to previous data, it was demonstrated that clinical pregnancy rate (CPR) was 50% lower in patients that have UC ≥ 4/min on ET day [26,32]. If uterine peristalsis ≥4/min was detected, i.v bolus dose of 6.75 mg atosiban (Tractocile; Ferring Pharmaceuticals, Switzerland) injection was performed in the AC-FET group before thawing. If UP continues 1 h later, the thawing procedure was not performed and ET was cancelled. ET was performed 2 h after the thawing procedure if UP did not continue. The number of transferred embryos was decided according to Turkish Health Ministry restrictions as mandatory single ET and conditional two ET rule. None of the patients were given adjuvant treatments.

Primary outcome was live birth rate (LBR). Secondary outcomes were CPR, implantation rate (IR), cycle cancellation rate, and endometrial thickness. Implantation rate was calculated by dividing the total number of gestational sacs on ultrasound by the total number of embryos transferred. Pregnancy test was performed by measuring serum βhCG level at 12 days after embryo transfer (ET) and intrauterine pregnancy was confirmed by using TVUSG 2 weeks after a positive pregnancy test. Clinical pregnancy was defined as intrauterine positive fetal cardiac activity in the ultrasound examination. LBR was defined as the birth of a fetus older than 32 weeks.

We did not perform a power analysis due to the retrospective design of our study. In a power analysis we performed for a future prospective study, the sample size computed by an online analyzer https://clincalc.com/stats/samplesize.aspx demonstrated that for an expected difference of incidences between the groups (20% vs 40%) with 5% level of significance and a power of 80%, a total of 162 patients (81 patients per group) were required. All statistical analyses were performed using SPSS v. 21 (IBM SPSS Statistics for Windows, Version 21.0. Armonk, NY: IBM Corp). Continuous variables with nonnormal distribution were presented as median (min–max) and compared with the Mann–Whitney U test. Fisher’s exact test was used for comparison of categorical variables; the chi-squared test was used for comparison of implantation rates. p-values <0.05 were considered to be statistically significant.

## 3. Results

Thirty-four patients with previous ET cancellation due to UP were included for final analysis. Endometrium was prepared for ET with artificial cycle that contains estradiol and progesterone supplementation (n: 23) and with stimulated cycle that contains letrozole (n: 11) (Figure). The baseline characteristics of each group were presented in Table 1. The groups were similar with respect to age, body mass index (BMI), duration of infertility, antral follicle count, and number of prior in vitro fertilization attempts. 

**Figure:**
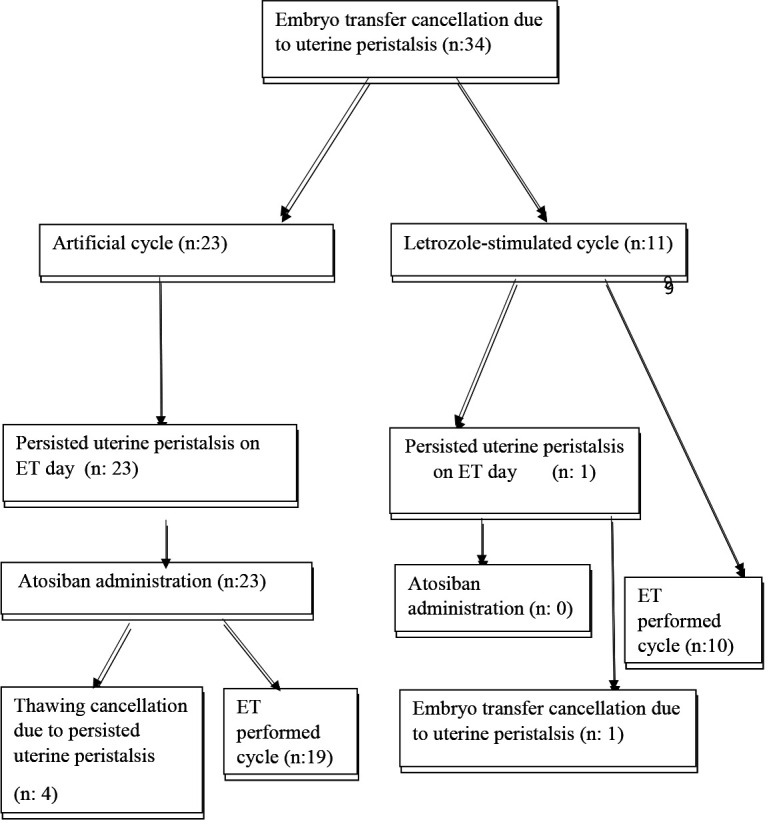
Flow diagram of patients with uterine peristalsis, atosiban administration, thawing cancellation, and embryo transfer (ET).

**Table 1:** Comparison of patient characteristics between the AC-FET and L-FET groups.

Variables	AC-FET group(n:23)	L-FET group(n:11)	p-value
Age (years)	29 (20–41)	32 (24–42)	NS
BMI(kg/m2)	23.6 (22.3–24.5)	24.2 (22.9–24.4)	NS
AFC (n)	15 (5–20)	15 (8–16)	NS
Duration of infertility (years)	4 (1–13)	4 (1–20)	NS
Prior IVF attempts (n)	3 (1–7)	2 (1–8)	NS
Etiology of infertility (n,%) Unexplained infertility Male infertility Tubal factor Mixed	12 (52%)7 (30%)2 (8.6%)2 (8.6%)	6 (54%)3 (27%)1 (9%)1 (9%)	
Freezing indications (n,%)Premature progesterone riseOHSSRemaining embryo	7 (30.4%)5 (21.7%)11 (47%)	3 (27%)2 (18%)6 (54%)	

The cycle characteristics and outcomes are presented in Table 2. Progesterone level on cycle day 2, endometrial thickness on ET day, and number of top quality embryos transferred were similar between two groups. The peak estradiol level (184 [150–316] vs 250 [150–470], p < 0.05) was significantly lower in the L-FET group as compared to the AC-FET group. Atosiban was administered in all patients of the AC-FET group due to UP that recurred in proceeding cycle and persisted on planned ET day. Atosiban administration rate was 100% in the AC-FET group and 0% in the L-FET group. Four cycles in the AC-FET group were cancelled due to UC ≥ 4/min despite atosiban injection on planned ET day. One cycle that had UC ≥ 4/min on planned ET day in the L-FET group were cancelled without atosiban injection. In the remaining patients, ET procedure was performed. The implantation rates (55.5% vs 11.7%); CPR per ET (90% vs. 15.8%); LBR per ET (70% vs. 5.3%) and CPR per cycle (81% vs. 13%); LBR per cycle (63% vs. 4.3%) were significantly higher in the L-FET group as compared to the AC-FET group. Cycle cancellation rates (9% vs 17.4%) were significantly lower in the L-FET group when compared to the AC-FET group. 

**Table 2:** Cycle characteristics and outcomes of each group.

Variables	AC-FET group	L-FET group	p-value
No. of top-quality embryos transferred (n) a	2 (1–2)	2 (1–2)	NS
Endometrial thickness(mm) a	10.9 (8–15)	10 (10–14)	NS
Progesterone level on the day of cycle day 2 (ng/mL)a	0.21 (0.05–0.76)	0.11 (0.05–0.23)	NS
Peak estradiol level (pg/mL)a	250 (150–470)	184 (150–316)	0.014
Cycle cancellation rate b	17.4% (4/23)	9% (1/11)	<0.001
Implantation rate (%,n/n) c	11.7% (4/34)	55.5% (10/18)	<0.001
Clinical pregnancy rate per ET (%,n/n) b	15.8% (3/19)	90% (9/10)	0.01
Live birth rate per ET (%,n/n) b	5.3% (1/19)	70% (7/10)	<0.001
Clinical pregnancy rate per cycle (%,n/n) b	13% ( 3/23)	81% (9/11)	<0.001
Live birth rate per cycle (%,n/n) b	4.3% (1/23)	63% ( 7/11)	<0.001

## 4. Discussion

Letrozole-stimulated endometrial preparation is an option for FET cycles especially in patients with ovulatory dysfunction [6,29,31]. To the best of our knowledge, this is the first study that investigates the potential advantages of letrozole in patients with a previous cycle cancellation due to uterine peristalsis and compares it with artificial cycle in terms of live birth rate. The present study demonstrated statistically significantly improved IR, CPR, and LBRs in L-FET cycles when compared with AC-FET cycles in this patient group. The difference in the cycle outcomes can be attributed to the increased endometrial receptivity due to decreased UP in the L-FET group while the numbers of top-quality embryos transferred were similar between groups. 

The underlying cause of implantation failures are impaired endometrial receptivity in two-thirds of cases and embryo quality in one-third [33]. Implantation is a crosstalk between endometrium, embryo, and maternal immune system [34,35]. An appropriate endometrial thickness and pattern, a sufficient blood flow [8,10–12] and a silent uterus [13–15,36] are needed for a receptive endometrium. It was shown that CPR was 50% lower in patients with UP ≥ 4/min than patients that have UP < 4/min and success rates of IVF/ET decrease 3-fold in cases of pronounced uterine contractions [26]. In our study, we selected a period of 1 min for assessment of UP according to previous data. This period was sufficient for detecting profound UP without any patient discomfort. The direction of UP was not noted as there was not any evidence that has shown its impact on implantation [26,32]. 

Uterine contractile activity is essential for reproduction. UP from fundus to cervix is needed to empty uterine cavity in early follicular phase. In the late follicular and periovulatory phases, UP from cervix to fundus is needed for sperm transport. In luteal phase, uterus becomes a relatively silent place for embryo implantation [27,37]. However, in ovarian stimulation cycles, both the catheterization of uterus during ET [38] and elevated estradiol levels can trigger excessive uterine contractions that can expel embryos from the uterus and decrease the pregnancy rates [15,26,39,40]. In the absence of uterine mechanical manipulation and uterine disorders like endometriosis, adenomyosis, leimyoma, and polyp; the mechanism underlying UP is exaggerated oxytocin action that is stimulated by elevated levels of estradiol [20,27].

Several medical treatments such as progesterone, anticholinergic agents, prostaglandin synthetase inhibitors, beta adrenergic receptor antagonists, and oxytocin receptor antagonists have been used for UP treatment [27]. It was demonstrated that progesterone, anticholinergic agents, and oxytocin receptor antagonists reduce UP in observational studies [27]. Atosiban is a combined oxytocin/vasopressin V1A antagonist that is used in the management of preterm labor. It is an effective, well-tolerated [41], and embryo-safe drug [42]. It was first used in IVF in a patient with recurrent implantation failure and pregnancy was documented as a case report in 2007 [43] and then the same author demonstrated that atosiban reduces uterine contractility and intrauterine prostaglandin F2α production and increases uterine blood supply [20]. Moraloglu et al. reported significant improvement in IR and CPR in atosiban group [44]. However, in a larger sample sized randomized double blind placebo controlled study, it was demonstrated that atosiban given around ET did not improve LBR in a general IVF population [37]. In both of these studies uterine contractions were not measured. Lan et al. showed that atosiban usage improved IR and CPR in women with recurrent implantation failure undergoing an AC-FET cycle when compared to previous cycles of patients [45]. Unlike the previous mentioned studies, Lan et al. measured uterine contractions and showed significant decrease of uterine contractions after atosiban administration [45]. In all of these studies, atosiban was used 3 h around ET and the last dose was given 2 h after ET. In our study, we had used one dose of i.v. bolus 6.75 mg atosiban and ET was performed 2 h after thawing if UP did not continue. However, IR, CPR, and LBR rates remained significantly lower when compared to the L-FET group that does not have UP on ET day. The reason why we did not find any benefit of atosiban on pregnancy rates in AC-FET group may be the lower dose and duration of atosiban administration. Due to the short half-life of atosiban, beneficial effects on reduction of contractions may not last through the implantation period and so may not improve pregnancy rates. A prolonged atosiban infusion or adding other drugs as nonsteroidal antiinflammatories can be an option [37]. However, further randomized trials are needed for accurate conclusions. 

It seems to be more logical to prevent the stimulation of UP rather than treat it. A prolonged time of atosiban infusion is not feasible and cost-effective. As it is known, expressions of uterine oxytocin mRNA and uterine oxytocin receptors were ovarian hormone-dependent and increases with stimulatory effects of estrogens [5,21,22,24]. Therefore, it seems that the main goal must be maintaining lower levels of estradiol in FET cycles of previous UP cases. 

In FET cycles, mild ovarian stimulation (OS) with aromatase inhibitors (AI) as letrozole gains popularity nowadays. In a recent metaanalysis, it was demonstrated that OS with gonadotropin or AI had significantly higher clinical pregnancy and live birth rates when compared to AC [1]. It was suggested that mild OS with letrozole may overcome subtle defects in folliculogenesis, naturally support luteal phase via development of corpus luteum in patients with ovulation disorders [6,31], normalize the increased aromatase P450 mRNA expression that is related to poor IVF outcome, and improve endometrial receptivity [46,47].

Hu et al. reported that letrozole-stimulated cycles had significantly higher IR, CPR, and ongoing pregnancy rate per ET when compared with artificial and human menopausal gonadotropin (hMG) stimulated FET cycles of patients with PCOS. In this retrospective pilot study, peak estradiol levels were significantly lower in the letrozole group than those in hMG group [48]. In a prospective randomized study of 1230 cycles with ovulatory disorders showed that IR, CPR, and LBR were significantly higher in the L-FET group when compared to the AC-FET but similar with those of the natural cycle (NC) group. Moreover, significantly lower abortion rate and estradiol level on human chorionic gonadotropin (hCG) day were also observed in the L-FET group [6]. Zhang et al. also demonstrated increased LBR in the L-FET group when compared to the AC-FET group in women with polycystic ovary syndrome in a retrospective study [29]. Lastly, CPR was significantly higher and E2 levels on ET day were significantly lower in the L-FET group compared the AC-FET group in a prospective randomized study in patients with normal menses [49]. In accordance with these studies, we demonstrated statistically significantly improved IR, CPR, and LBR with a lower peak E2 levels and cancellation rates in L-FET cycles. Differently, we had a study population of patients with a history of UP-related cycle cancellation. Promising results of letrozole on pregnancy outcomes and E2 levels encourage us for the usage of letrozole in patients with UP history. 

Limitations of our study were its retrospective nonrandomized design and small sample size. Since the number of patients in the groups of our study is low, it is a possibility that statistical outcomes are by chance. However, it is difficult to get enough patients that have UP in a single center. The evaluation method of UP might be a limitation. The number of UP per minute was calculated as the number of peristaltic waveforms seen directly in the endometrium in sagittal plane on transvaginal ultrasound examination for 1 min. Although the counting of visible waves seems to be subjective, the number of peristaltic waveforms could be clearly noticed throughout the endometrial cavity and it could be clearly counted in patients. Due to the absence of a computer-assisted image analysis system in our clinic, we had to select this method. Another limitation was the usage of atosiban only in the AC-FET group due to UP and there was not any control group for atosiban in the L-FET group. The fact that the embryo transfer days are not similar is another limitation for this study. It was the advantage that LBR was the end point.

In conclusion, letrozole seems to be a promising protocol for the patients with a history of UP. However, large sample sized randomized studies are needed to reach certain conclusions.

## Ethical approval

The institutional review board and ethics committee approved the study with the number 2020/426.
